# Dynamical Disorder in the Mesophase Ferroelectric HdabcoClO_4_: A Machine-Learned Force Field Study

**DOI:** 10.1021/acs.jpcc.4c06615

**Published:** 2024-12-18

**Authors:** Elin Dypvik Sødahl, Jesús Carrete, Georg K. H. Madsen, Kristian Berland

**Affiliations:** † Department of Mechanical Engineering and Technology Management, Norwegian University of Life Sciences, N-1433 AS, Norway; ‡ Instituto de Nanociencia y Materiales de Aragón (INMA), CSIC-Universidad de Zaragoza, ES-50009 Zaragoza, Spain; § Institute of Materials Chemistry, TU Wien, A-1060 Wien, Austria

## Abstract

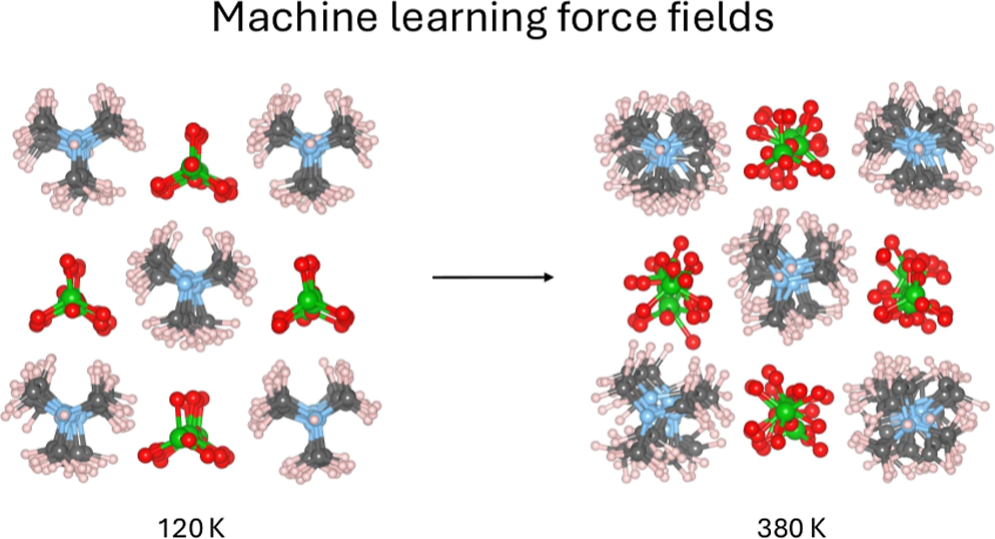

Hybrid molecular ferroelectrics with orientationally disordered mesophases offer significant promise as lead-free alternatives to traditional inorganic ferroelectrics owing to properties such as room temperature ferroelectricity, low-energy synthesis, malleability, and potential for multiaxial polarization. The ferroelectric molecular salt HdabcoClO_4_ is of particular interest due to its ultrafast ferroelectric room-temperature switching. However, so far, there is limited understanding of the nature of dynamical disorder arising in these compounds. Here, we employ the neural network NeuralIL to train a machine-learned force field (MLFF) with training data generated using density functional theory. The resulting MLFF-MD simulations exhibit phase transitions and thermal expansion in line with earlier reported experimental results, for both a low-temperature phase transition coinciding with the orientational disorder of ClO_4_^–^ and the onset of rotation of both Hdabco^+^ and ClO_4_^–^ in a high-temperature phase transition. We also find proton transfer even in the low-temperature phase, which increases with temperature and leads to associated proton disorder as well as the onset of disorder in the direction of the hydrogen-bonded chains.

## Introduction

Hybrid molecular crystals and salts have recently attracted much interest due to their vast potential application range, including as electrolytes,^1−3^ barocaloroics,^4^ piezo-, and ferroelectrics.^5−10^ Moreover, the possibility of using room-temperature synthesis with low-energy methods, such as 3D-printing,^11^ slow evaporation,^12−14^ and spin coating,^15^ allow for environmentally friendly production and flexible device integration. As different molecular species can be combined in many ways, they offer immense design flexibility, which can circumvent the need for toxic molecules and/or scarce elements. Some of these molecular crystals and salts, especially those consisting of globular (i.e., cage-like, disk-like, or cylindrical)^3,6^ molecules can host plastic mesophases where the molecular species become orientationally disordered while retaining crystalline order.^16^ The onset of the orientational disorder, can also result in a marked increase in the number of facile slip planes, contributing to the possibility of fusing or molding the molecular crystals into desired shapes and this class of materials are therefore often referred to as plastic crystals.^6,17^

The degree of disorder can vary between plastic crystals, and some also display transitions between partly and fully orientationally disordered mesophases.^15,18,19^ The large entropy change in the transition from an ordered low-temperature phase to a disordered plastic phase^20−23^ can also be used for thermal storage and barocaloric cooling applications.^4,24^ These materials also have potential as effective ionic conductors.^1−3^ For ferroelectric plastic crystals, the transition to the plastic mesophase often coincides with a transition to a paraelectric phase.^5,25−28^

Molecular components of plastic crystals include neutral species such as paraffins and cycloalkanes,^6^ cationic species such as derivates of quinuclidine, dabco (1,4-diazabicyclo[2.2.2]octane), and tetramethylamine,^5,7^ and anionic species such as ClO_4_^–^, FeCl_4_^–^, OCN^–^, and H_2_PO_4_^–^.^1,5^ Recently, we attempted to uncover novel ferroelectric molecular crystals from the Cambridge Structural Database^29,30^ finding 20 new systems that are likely to be both ferroelectric and plastic crystals.^30^

Although many properties of plastic crystals have been characterized, a microscopic understanding of the phase transitions and the nature of the disorder in plastic crystals is still largely missing. There is also limited insight into the polarization-switching mechanisms of these materials.^7^ Such insight can be provided by molecular dynamics (MD) simulations; however, parametrizing classical force fields can be nontrivial, particularly for systems with a complex bonding nature such as the hybrid ionic crystals.^31^ As the bonding picture can include charge transfer, highly anharmonic vibrations, hydrogen bonding, and in some cases proton transfer, it may be hard to ensure that the specific functional form of the interaction potentials well describes all salient chemical effects of a system.^32^ Ab initio molecular dynamics, on the other hand, compute all electronic bonding effects, typically at the density functional theory (DFT) level.^33^ While this approach can provide much insight into smaller systems, computational costs can become prohibitive for typical plastic crystals at the relevant time scales and supercell sizes. The recent advent of machine-learned force fields (MLFFs) that can be trained on ab initio data has opened the door for predictive modeling of dynamic materials, which with sufficient diverse data can approach the accuracy of the underlying DFT-based training data.^32,34−40^

**Figure 1:**
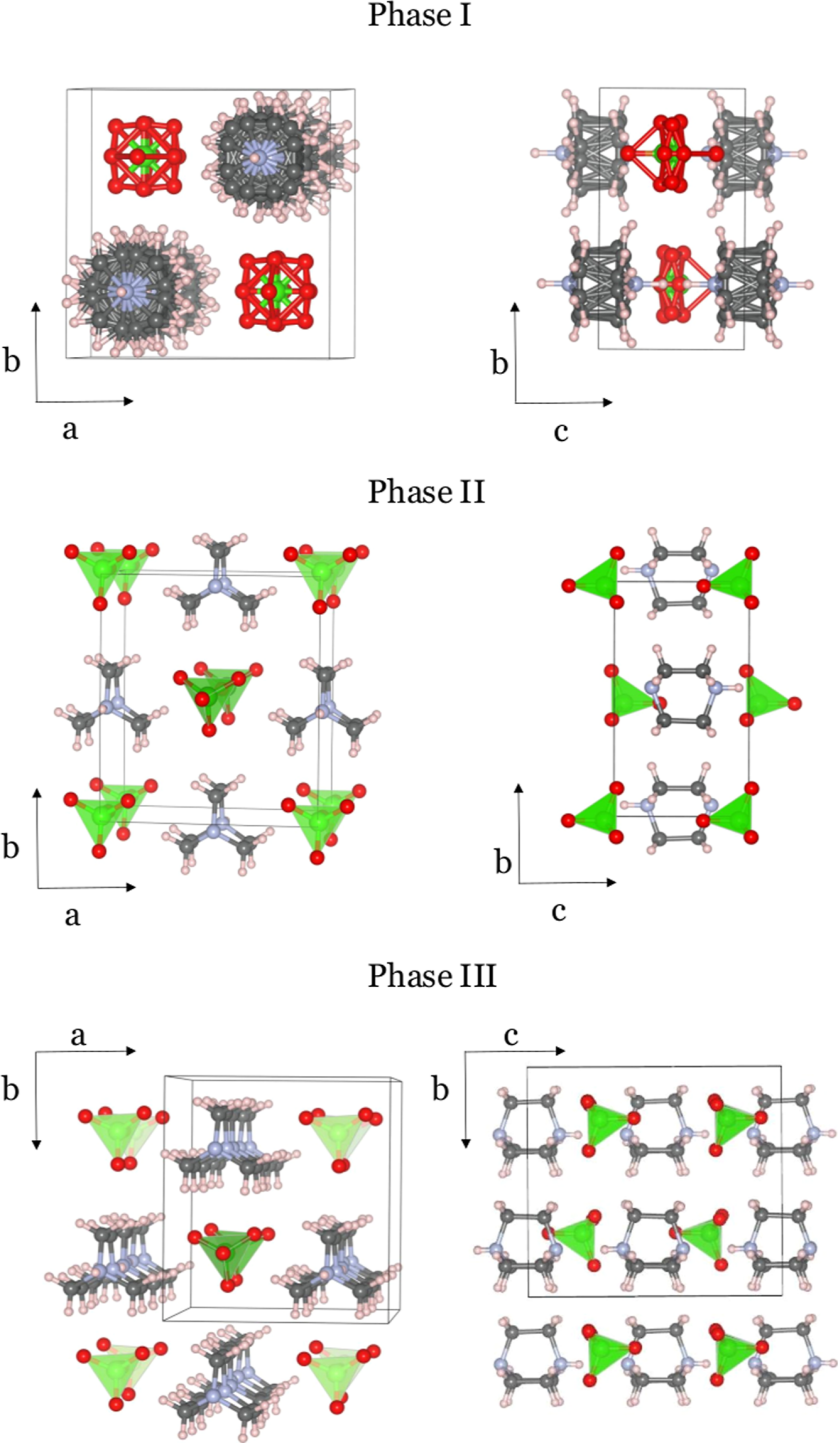
Illustration of the reported experimental crystal structures of phases I, II, and III of HdabcoClO_4_. The atoms are indicated by the following colors, C: gray, N: blue, O: red, Cl: green, H: white.

In this work, we used such an approach to study phase transitions and dynamical properties of HdabcoClO_4_ (1,4-diazabicyclo[2.2.2]octan-1-ium perchlorate) in the 120–500 K range. Rather than a rotational switching mechanism, exhibited by many reported ferroelectric plastic crystals,^41,42^ HdabcoClO_4_ has a displacive-type ferroelectric switching, which makes it capable of ferroelectric switching at frequencies up to 10 kHz.^43^ It has a spontaneous polarization of 4.6 μC/cm^2^ and a Curie temperature of 377 K^44−46^ which is quite large for this class of compounds. The material also exhibits a rich phase diagram, dynamical disorder, and a partially orientationally disordered mesophase,^18,43−47^ with nine phases reported below the decomposition temperature at 535 K.^44^Figure 1 displays the structure of the paraelectric mesophase I, the room-temperature ferroelectric phase II, and the low-temperature ferroelectric phase III. In all the phases, Hdabco^+^ form hydrogen-bonded columns. In the high-temperature phase I, partial proton disorder has also been reported. Whereas the ClO_4_^–^-anions have a large degree of oriental disorder in the mesophase, the Hdabco^+^-cations predominantly show disorder around the hydrogen-bond direction.

## Methods

### Density Functional Theory

The DFT calculations were based on the plane-augmented wave (PAW) formalism^48,49^ as implemented in the Vienna Ab initio Simulation Package (VASP).^50−53^ The nonlocal van der Waals density functional vdW-DF-cx^54−56^ was selected as it can provide accurate lattice constants of highly diverse solids,^56−58^ and we recently found it to provide accurate lattice constants of several plastic crystals.^29,59^ The DFT simulation cells were based on 2 × 2 × 2 times the unit cell of phase II, corresponding to a cell with 416 atoms in total. In the DFT-MD simulations, the plane-wave cutoff was set to 530 eV using a Γ-point sampling of the Brillouin zone. All DFT-MD simulations started from relaxed until cells. The DFT-MD simulations were carried out under the action of a Nóse–Hover thermostat^60,61^ and an *NVT* ensemble with a time step of 0.5 fs.

### Machine-Learned Force Field: Training and Simulations

The machine-learned force field was trained using the neural-network-based NeuralIL^36^ employing a Residual Neural network (ResNet) framework^62^ implemented on top of Jax^63^ and Flax.^64^ In this method, the local environment within a radius of *r*_cut_ of an atom is decomposed into spherical Bessel descriptors.^65^ Using a local coordinate system ensures translational invariance, while rotational invariance is ensured by using the scalar power spectrum of the projections over basis functions. The chemical identity of the central atom is accounted for with embedding coefficients given by the type of element. The descriptors and embedding coefficients are in turn fed into the neural network.^36^ The core widths of the ResNet were set to 64:32:16. We used a batch size of eight and trained the MLFF for 25 epochs, which are sufficient thanks to the highly efficient nonlinear optimizer VeLO.^66^ In the training of the MLFF, we set the weight of the energy to 0.4 and the remaining 0.6 was assigned to forces. A radial cutoff of *r*_cut_ = 4.0 Å was selected, based on a convergence study increasing the *r*_cut_ from 3.5 to 5.0 Å in steps of 0.5 Å, until we found no further reduction in error in the validation set. Similarly, the maximum radial order of the basis functions for the spherical Bessel descriptors was set to 4. For the committee-based active learning, ten models were used to provide an uncertainty metric in the MLFF.

The MLFF was trained in four stages. First, a crude model was trained using selected DFT-MD data. Second, we iteratively included additional DFT-MD data in six steps by explicitly comparing the prediction error between the model and the DFT computations. Third, we used a committee-based active-learning procedure to obtain a more diverse training set and a stable MLFF. In the final step, we added training data in which the unit cells were compressed and expanded, as well as data where atomic species were swapped to alter the chemistry. This step provided further diversification of the training set and ensured that highly unfavorable configurations were represented in the data. 20% of the configurations in the training set were randomly set aside for validation in each iteration of the training.

Initial DFT-MD simulations were carried out at different temperatures and volumes to obtain diverse yet physically representative starting training data, as shown in Table 1. For simulation C, one ClO_4_^–^ anion and one Hdabco^+^ cation were manually rotated in the initial configuration to force molecular rotations during the simulation. The first MLFF model was based on 400 configurations randomly selected from DFT-MD simulations C and D. Although this produced an MLFF with low root-mean-square errors (RMSE) for both forces and energies for the validation set, 99 meV/Å and 3.0 meV/atom, this model was inherently unstable, exhibiting cell “explosions” at 300 K. In the next step, 50 configurations from all sets of DFT-MD data were added in each iteration based on the largest deviations in force predictions between the MLFF and the DFT-MD data, as shown in Table 2. Despite significantly reduced validation errors within the expanded training sets, subsequent MD simulations still resulted in unstable cell volumes for temperatures above 300 K. This illustrates that relying only on DFT-MD data to train an MLFF can be insufficient, as the short time scales feasible can be insufficient for providing sufficiently diverse training sets.

**Table 1:** DFT-MD Training Data

	temperature (K)	volume	
A	400	*V*_relax_	
B	600	*V*_relax_	
C	600	*V*_relax_	forced mol. rot
D	600	0.98 × *V*_relax_	
E	600	1.02 × *V*_relax_	
F	800	*V*_relax_	
G	800	0.9 × *V*_relax_	

**Table 2:** Overview of MLFFs Trained on Selected DFT-MD Data^a^

configs.	*F*_RMSE_ (meV/Å)	*E*_RMSE_ (meV/atom)	Δ*F*_max_ (eV/Å)
400	99	3.0	176
450	136	5.9	6.79
500	113	7.3	5.73
550	900	4.9	2.63
600	100	8.3	3.19
650	147	5.4	8.23
700	112	4.6	

a *F*_RMSE_ and *E*_RMSE_ are the errors computed for the validation set. Δ*F*_max_ is the maximum deviation between the MLFF and DFT-MD predicted forces.

In the committee-based active learning, ten MLFFs were trained using the same training set. Using the implementation of Carrete et al.,^36^ all ten were trained in the same run with different initial random coefficients. New atomic configurations were generated by running an MD simulation with a duration of 50 ps using the MLFF model only trained on DFT-MD data. 1000 configurations were evenly sampled and used as input for the committee. The standard deviation in the force predictions for the predictions of the committee was then used to identify atomic configurations that were not represented in the training set. Next, DFT computations were performed for the 200 configurations with the largest standard deviations in forces, and the configurations were added to the training set. A new MLFF was then trained, and the procedure was repeated four times as shown in Table 3. The first three iterations used MLFF-MD simulations at 300 K and 1 bar. The fourth training set combined several MLFF-MD simulations at 400 and 800 K with pressures ranging from 1 bar to 9 kbar as input to the committee. After the fourth iteration, the volume predictions stabilized, and the largest standard deviation in the volume was found for a simulation at 450 K with a value of /formula unit.

**Table 3:** Overview of MLFFs Trained using Active Learning Data and the Errors Computed from Validation^a^

configs.	*F*_RMSE_ (meV/Å)	*E*_RMSE_ (meV/atom)	σ_max_ (eV/Å)
900	100	3.2	121
1100	87	5.5	0.071
1300	114	2.4	0.005
1500	84	3.0	0.003

a *F*_RMSE_ and *E*_RMSE_ are the root square mean error in the forces on the configurations in the validation set. σ_max_ is the largest standard deviation computed in the active learning procedure.

Finally, the training data was further expanded to ensure that highly nonfavorable configurations were represented in the training of the force field. We used two approaches to achieve this. 480 configurations were constructed by scaling the unit cell parameters with a factor ranging from 0.9 to 1.1 in increments of 0.1. This was applied for each unit cell parameter individually, but also to the volume of the cell. This results in compressed and expanded unit cells where the molecular geometries differ from their relaxed geometry. In addition, we constructed 200 configurations in which two atoms in either Hdabco^+^ or ClO_4_^–^ swapped positions. This ensured that less favorable chemistry was represented in the training data and can thus be appropriately avoided in the MD simulations. The forces and energies for all configurations were computed using DFT, and the final training set then contained 2180 configurations. The resulting model had validation errors of *F*_RMSE_ = 89 meV/Å and *E*_RMSE_ = 4.5 meV/atom.

MD simulations using the MLFF were performed using Jax-MD,^67^ with a time step of 0.25 fs. Thirty ps were used for thermalization, and the production runs were 180 ps. An *NPT* ensemble was used with a Nosé–Hoover chain thermostat^68^ and a barostat^69^ allowing flexible simulation cells using the integrator suggested by Yu et al.^70^ as implemented by Bichelmaier.^71^ The pressure was fixed at 1 bar in all simulations. The simulations were initialized from simulation cells based on 6 × 6 × 6 times of the unit cell of phase II of HdabcoClO_4_, which corresponds to a supercell size of 52.7 × 58.6 × 32.1 Å, containing 11,232 atoms or 432 ionic pairs of ClO_4_^–^ and Hdabco^+^. In total, 19 simulations with fixed temperatures in the range between 120 and 500 K were performed with a denser temperature sampling around the expected mesophase transition temperature.

## Results and Discussion

In the following, we discuss the thermal expansion, average displacement, and orientational disorder that arise in HdabcoClO_4_ at different temperatures.

### Thermal Expansion

**Figure 2:**
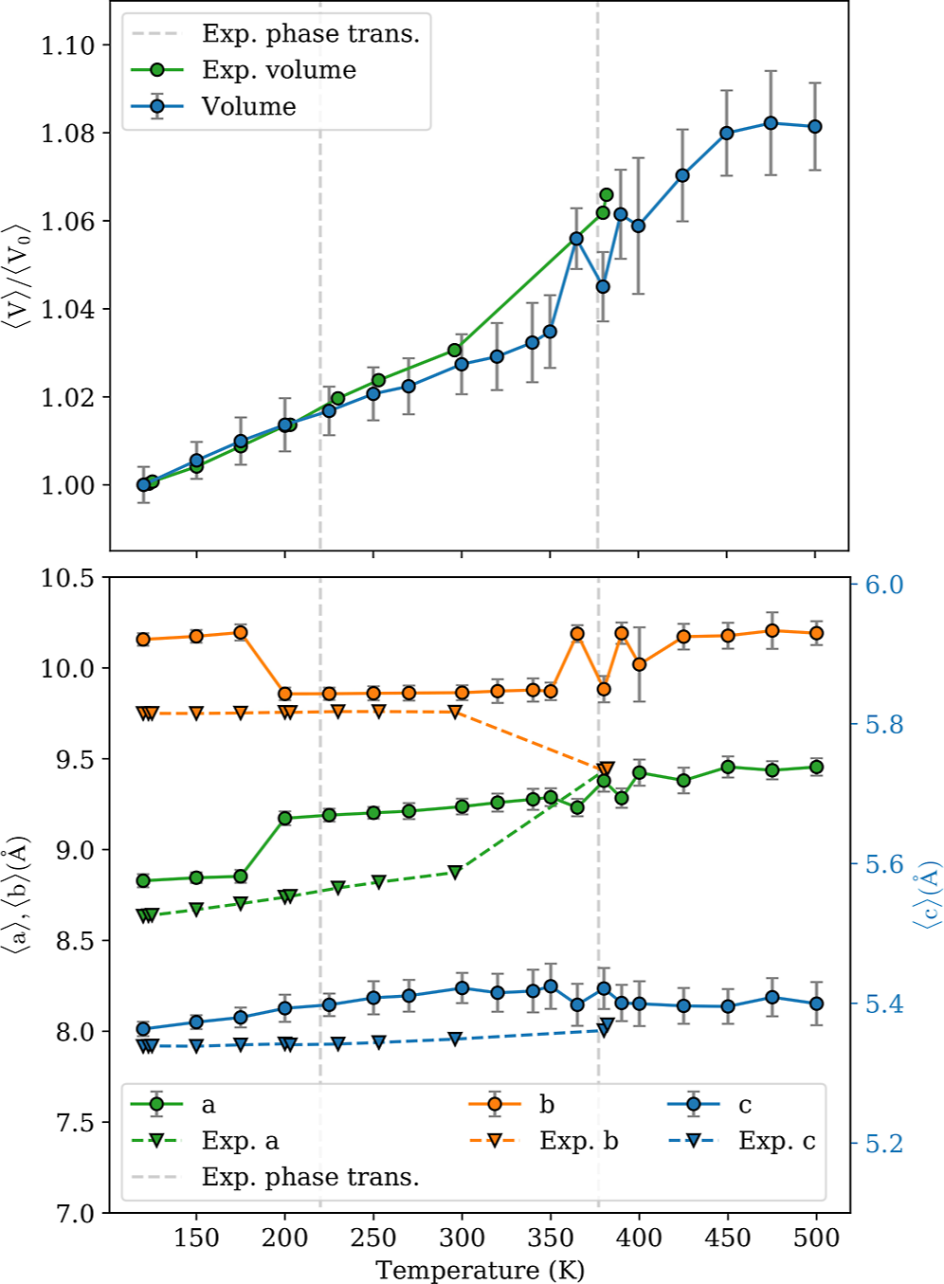
Normalized experimental^18,44,47^ and predicted volumes (top), and experimental and predicted cell parameters (bottom). The bar half-length denotes two standard deviations. The cell parameters correspond to the phase-II lattice. The vertical dashed lines mark the experimental phase transition temperatures.^44^

Figure 2 plots the computed and experimental volumes normalized to those at 120 K (*V*_0_) (top panel) and associated lattice constants (bottom). The computed volumes overestimate the experimental ones, by between 7% for temperatures up to 300 K and 5.5% at 380 K. The change in slope, i.e., the thermal expansion, from that below 350 K to that above 400 K, and the fluctuations in between is in line with the experimentally observed phase transition at 377 K.

In the bottom panel of Figure 2, the full lines indicate the computed lattice constants, given by 1/6 of the supercell lattice parameters, which correspond to the lattice constants of phase II. The hydrogen-bonded chains of Hdabco^+^ align with the *c* axis. The computed values of *c* agree well with experiment, with the largest deviation, an overestimation of approximately 1.3%, at 350 K. For *a* and *b*, the deviations are larger, up to 4.9% for both. The computed *a* and *b* values show anomalies at 200 K, where *a* increases and *b* decreases, which is not reported experimentally. At 380 K, the experiment shows *a* = *b*, which is not found in our MD simulations, where *b* instead exhibits a small, sudden increase. The larger deviations for *a* and *b* may be due to limitations in MLFF and the training procedure, or it could be due to the choice of the exchange–correlation functional. Although vdW-DF-cx is highly accurate at typical equilibrium distances,^59^ it tends to overestimate the interaction energies for dispersion-bonded molecular dimers beyond equilibrium.^58,72^ This overestimation could lead to overestimated lattice constants in phases characterized by dynamic disorder. However, the experimental observation *a* = *b* may also mask a more complex static or dynamic disorder occurring at longer length scales and time scales than what can be probed with our MD simulation, but which is averaged out in the experimental characterization.^73−75^

### Ionic Displacement and Spontaneous Polarization

**Figure 3:**
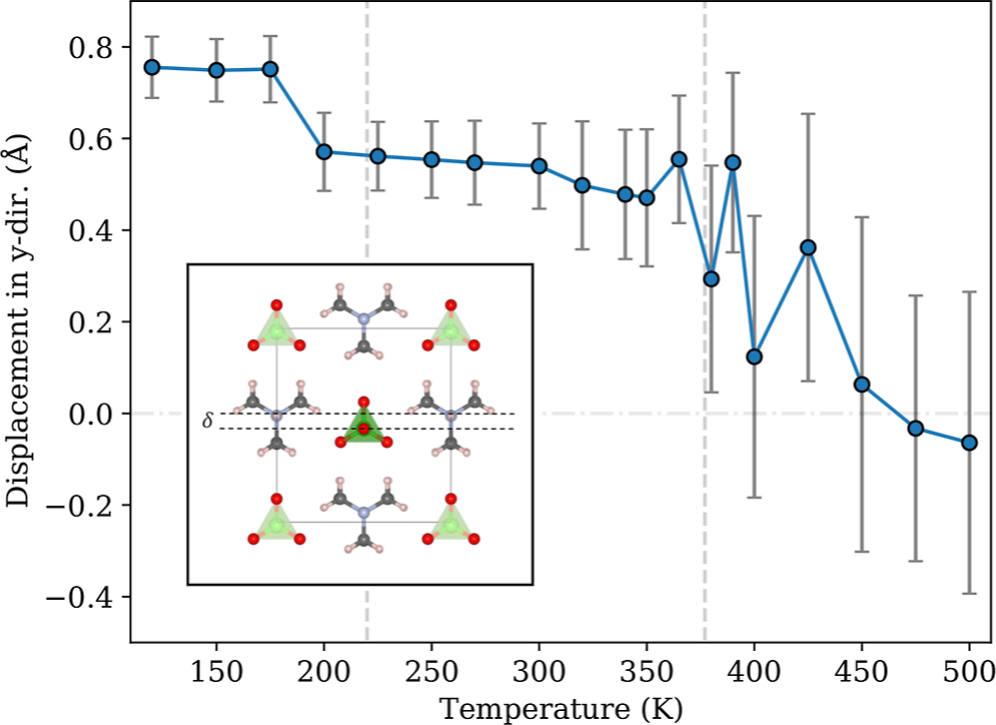
Average displacement ⟨δ⟩ of Hdabco^+^ cations relative to the ClO_4_^–^ anions within the same layer in the simulation cell, with two standard deviations indicated. The inset illustrates the displacement in phase II.

Figure 3 shows the average ionic displacement δ in the *b*-direction of Hdabco^+^ relative to the ClO_4_^–^ columns. This parameter is linked to the spontaneous polarization of HdabcoClO_4_^46^ and serves as a ferroelectric-to-paraelectric order parameter. For temperatures up to 175 K, δ ∼ 0.75 Å, before dropping to 0.58 Å at 200 K, coinciding with the transition between phases III and II. At 380 K there is also a marked drop in δ with a large increase in the corresponding deviations and further anomalous behavior before approaching low values beyond 450 K, but with large deviations. This is line with a broadened phase transition, where larger supercell sizes and/or longer time runs might result in sharp phase-transition temperatures.

### Orientational Disorder

#### ClO_4_^–^: Rotational Dynamics

**Figure 4:**
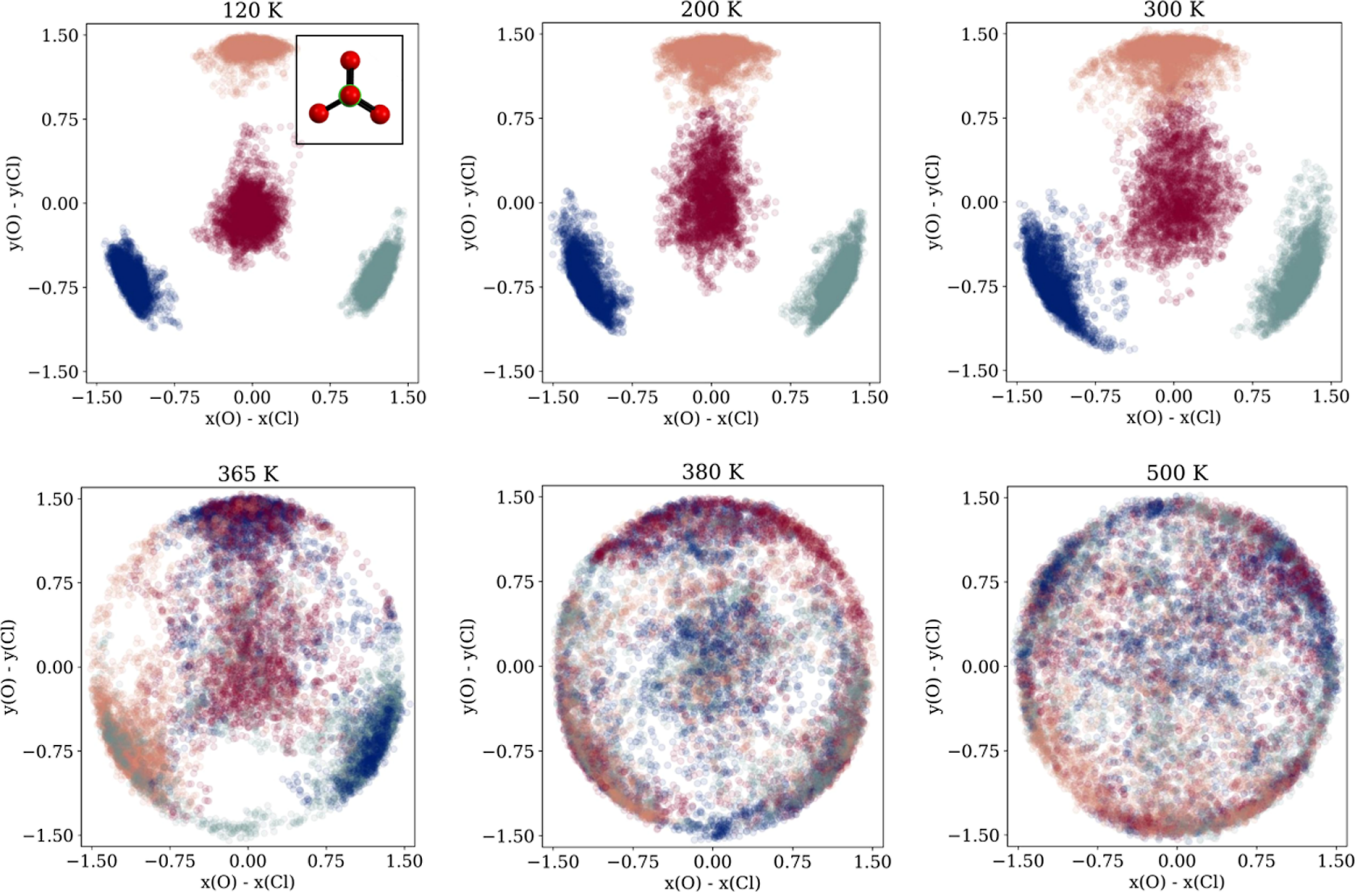
Oxygen atom positions relative to chlorine in a ClO_4_^–^ throughout the simulation, colored by their initial position in the ClO_4_^–^-cation. ClO_4_^–^ is viewed from the above as indicated in the inset.

Figure 4 plots the oxygen atom positions (four different colors) of a selected ClO_4_^–^ anion in the *x*–*y* plane relative to its central Cl atom throughout an MD simulation for temperatures ranging from 120 to 500 K. In the 120–300 K range, the plots show that the tetrahedron has a preferred orientation, with libration motion that increases with temperature. At 365 K, there is clearly a significant rotation as seen by the mixing of colors, but a distinctly preferred axis of orientation remains. This preference weakens at higher temperatures due to a transition into full rotational disorder in the *x*–*y*-plane, with also significant rotations on the sphere itself.

**Figure 5:**
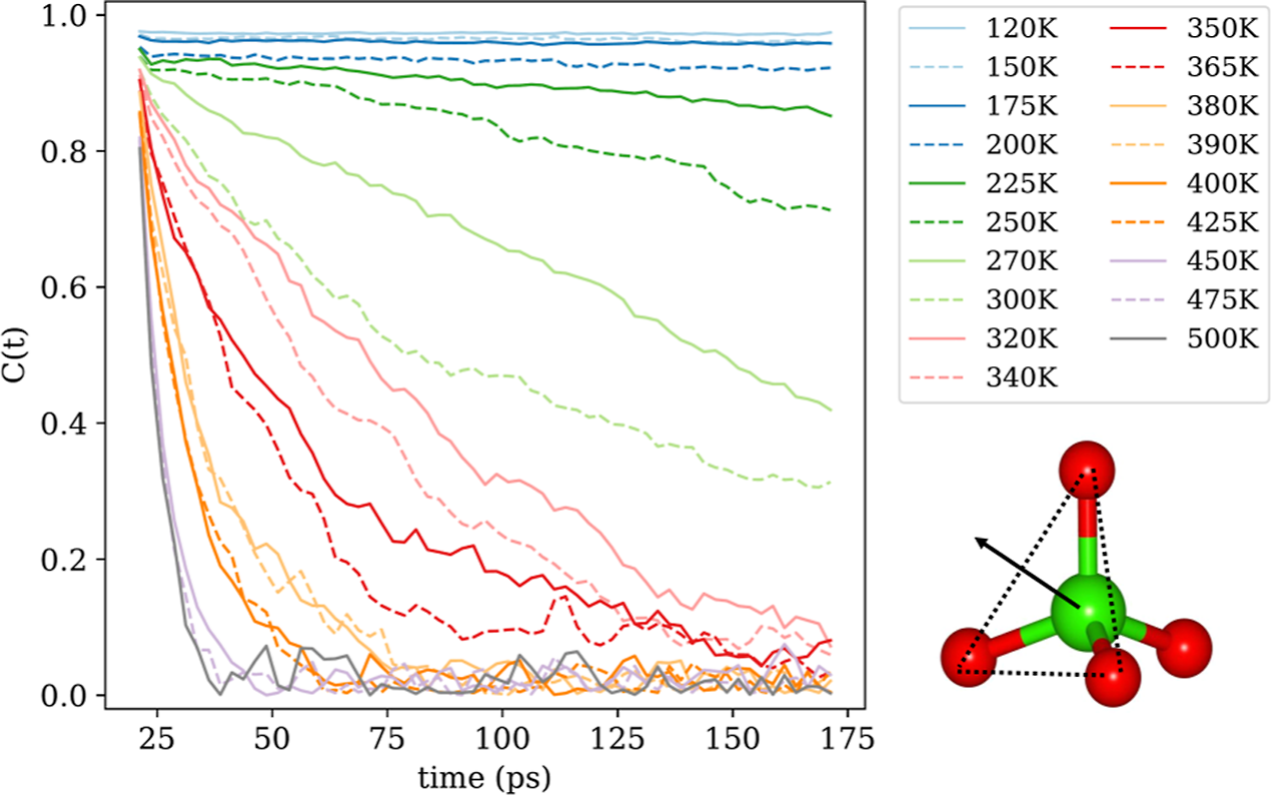
Rotational autocorrelation of ClO_4_ for different temperatures. The molecular direction for the autocorrelation is the vector from the chlorine atom to the center of a tetrahedral face, as illustrated to the right. The chlorine atom is shown as green and oxygen as red.

The rotational disorder of the ClO_4_^–^-anions is evaluated using a rotational autocorrelation function^19,76^
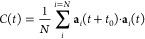
1where **a**_*i*_ is given by a unit vector pointing from the central Cl atom to the center of a tetragonal face spanned by oxygen atoms. Figure 5 plots *C*(*t*) for temperatures between 120 and 500 K. *C*(*t*) decreases with temperature but remains close to 0.9 for temperatures between 120 and 175 K, i.e., an indication that no rotation occurs during the simulation. At 200 K, *C*(*t*) begins to steadily decrease, indicating the onset of occasional rotation of ClO_4_^–^-anions, which increases with temperature. This finding is in line with a phase transition between phase III and II and the shift in δ found at this temperature in Figure 3. The very rapid decay of *C*(*t*) beyond 380 K compared to the more conventional exponential decay at lower temperatures is also possibly reflecting the phase transition occurring between 365 and 380 K.

### Hdabco^+^: Rotation and Tilting

**Figure 6:**
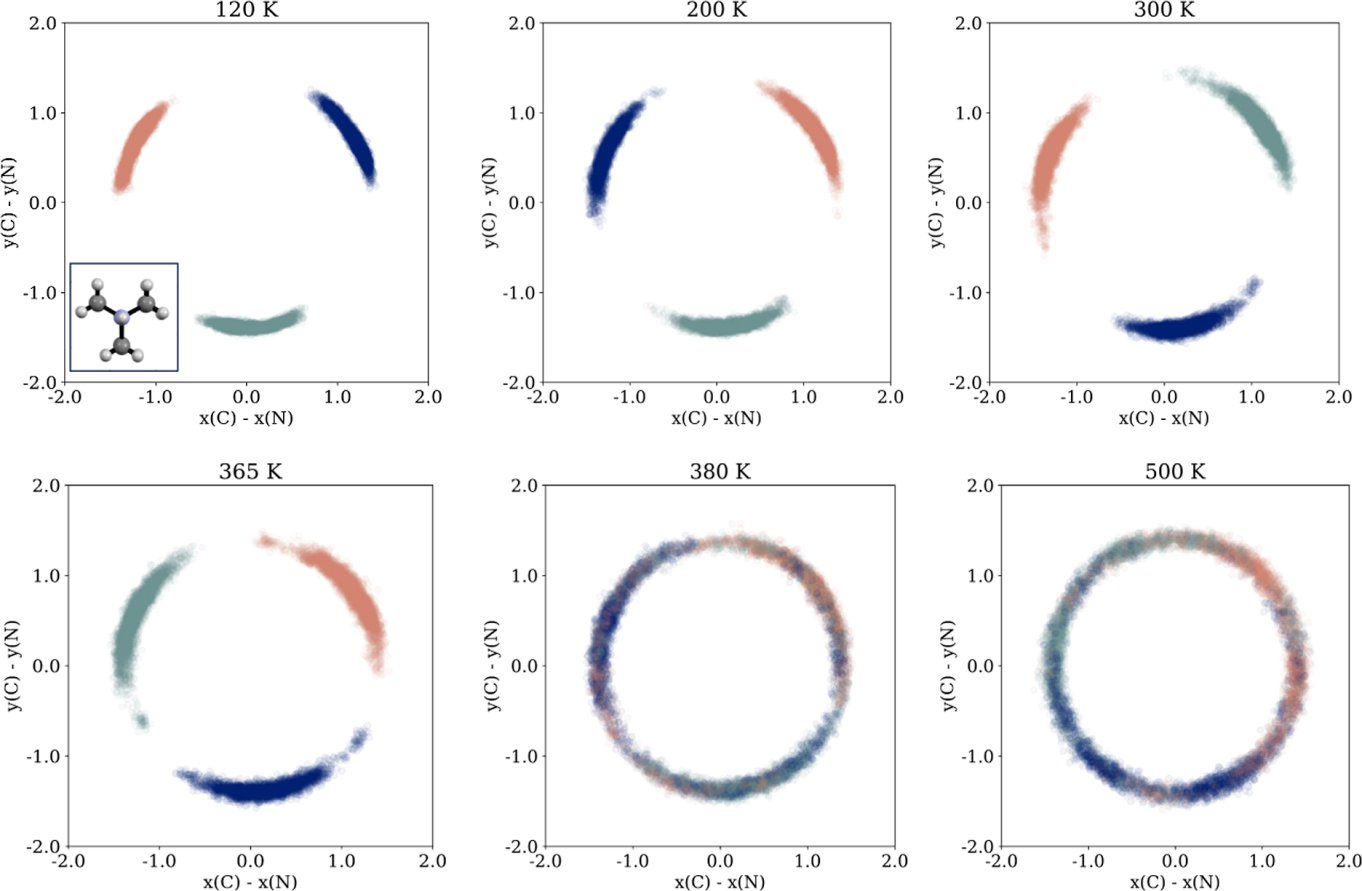
Illustration of the positions of three carbon atoms in a Hdabco-cation relative to their nitrogen neighbor atom. The three colors each represent a distinct carbon atom in the cation. The cation is viewed along the hydrogen-bonded direction, as illustrated in the inset. The onset of molecular rotation around the hydrogen-bonded axis is at 380 K.

For the Hdabco^+^-cations, we found rotation to only occur around the *c* axis. Figure 6 plots the carbon atom position relative to the N atom in the plane perpendicular to the *c*-axis, in the range of 120–500 K. Up to 365 K, the plot shows increasing libration with temperature, but no onset of rotation. At 365 K, there is a larger spread in the carbon atom position, and at 380 K and above, the trajectories indicate frequent rotations, in excellent agreement with the experimental phase transition temperature at 377 K.

The constrained rotation of Hdabco^+^ cations at elevated temperatures indicates that the hydrogen bonds are stable throughout the temperature range studied. This is also reflected in the volume expansion, as the length of *c*, the only hydrogen-bonded direction, is close to constant when temperature increases, even across phases. Hydrogen bonds have also been reported to be central to the mesophase behavior of plastic crystals. Yoneya and Harada^19^ studied quinuclidinium perrhenate using classical MD and found that a partially disordered phase was stabilized relative to the fully disordered mesophase, as intermolecular hydrogen bonds outcompete the thermal disorder for temperatures up to 367 K.

The onset of orientational disorder of ClO_4_^–^ and Hdabco^+^ coincides with phases II and I, respectively. A similar behavior was reported for tetramethylammonium dicyanamide by Adebahr et al.^76^ They used MD with classical force fields and identified the onset of rotation of each of the two molecular entities as the driving mechanisms for two distinct phase transitions of the material.

**Figure 7:**
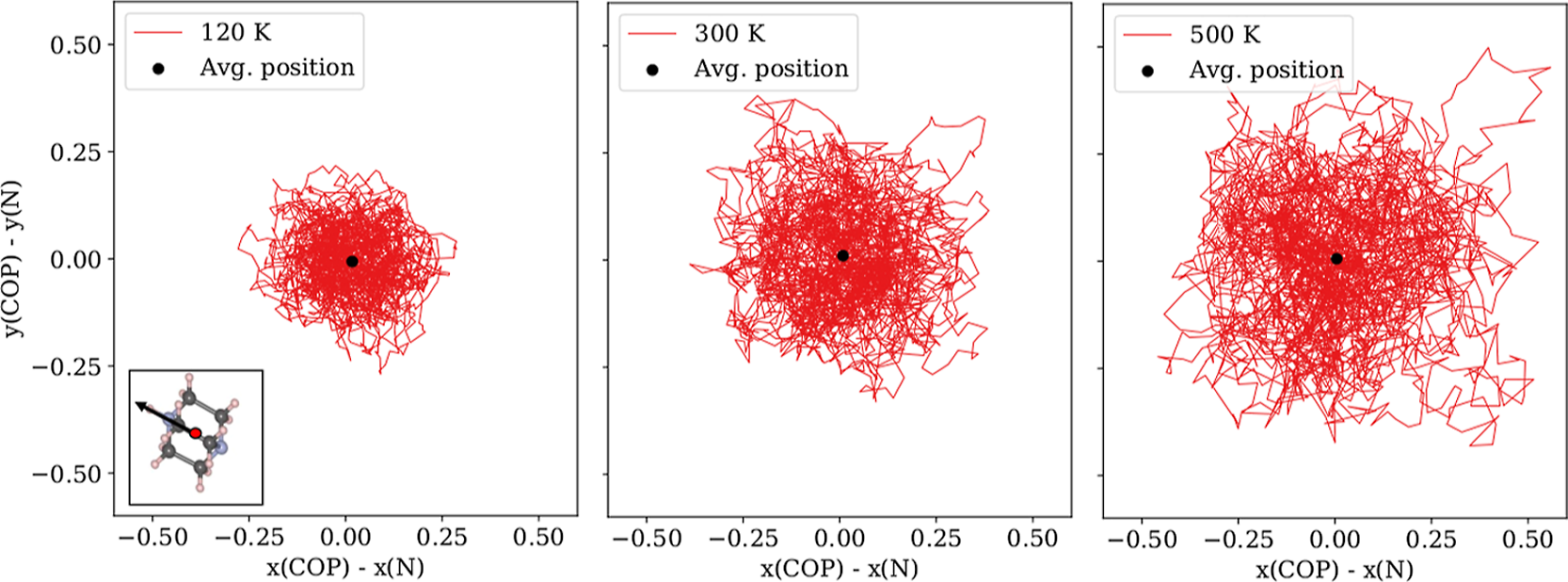
Position of the nitrogen atom in an Hdabco^+^-cation relative to the center of the positions of the cation, as illustrated in the inset (central position marked in red in the inset).

**Figure 8:**
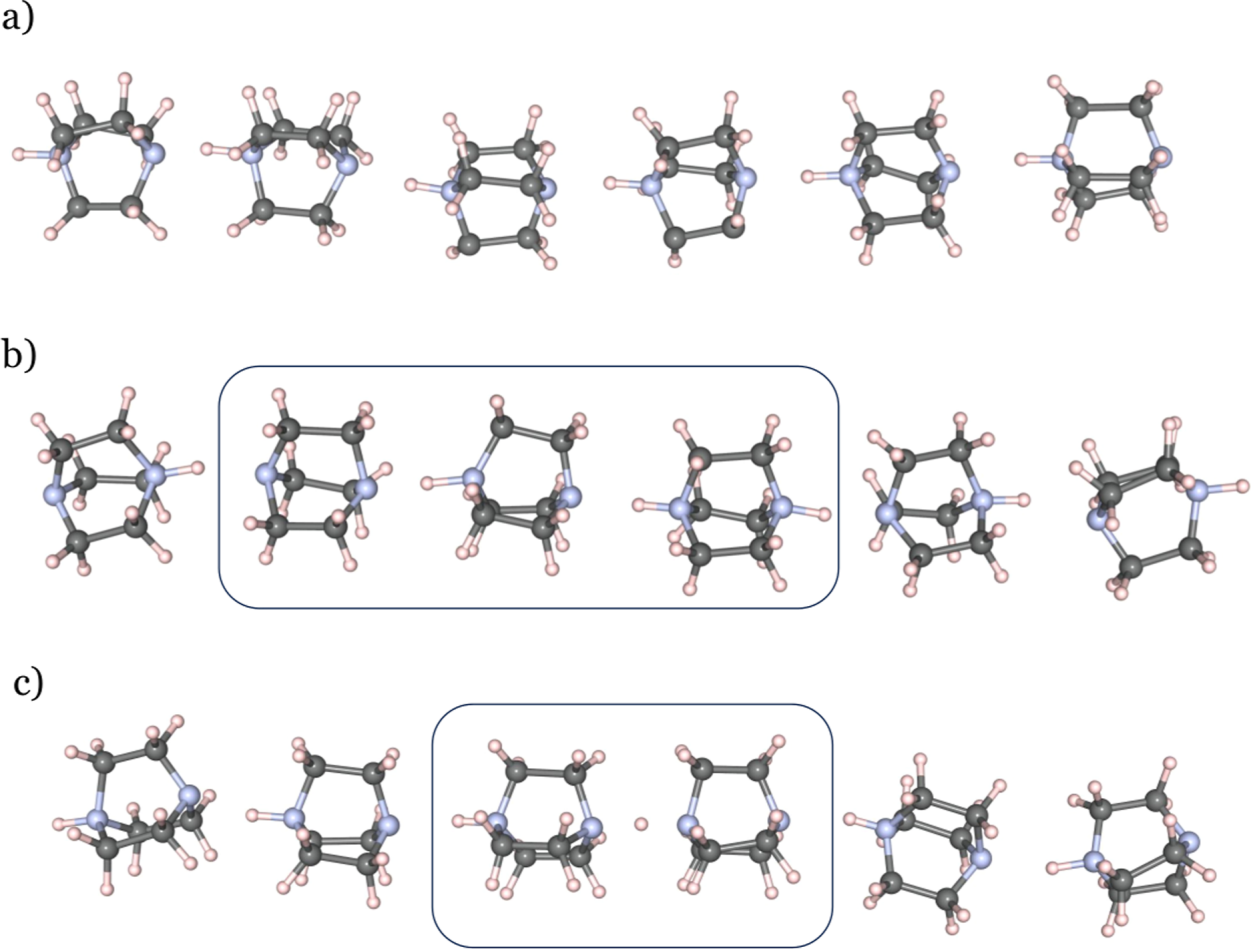
Snapshots of hydrogen-bonded chains at 425 K: (a) shows an aligned chain without defects, (b) a chain with a double-protonated and deprotonated dabco-molecule, and (c) a chain with a deprotonated molecule and a proton situated in the middle of two molecular species. Carbon atoms are shown in gray, nitrogen in blue, and hydrogen in white.

Figure 7 displays the position of one of the nitrogen atoms in a Hdabco^+^ cation relative to the center of positions of the cation during simulation, as illustrated in the inset. The variation shows that in addition to the rotation around this center, the tilt of the cations increases with temperature. Examples of the type of tilt the Hdabco^+^ cations exhibit relative to the direction of the chain are also shown in Figure 8, obtained at 425 K.

### Proton Disorder and Hydrogen Bonds

Proton disorder is also found in our simulations. Figure 8 displays hydrogen-bonded chains of Hdabco^+^-cations simulated at 425 K. Figure 8a illustrates a hydrogen-bonded chain of Hdabco^+^ cations at 425 K without defects, where all hydrogen bonds are oriented in the same direction. Figure 8b shows a case where the transfer of a proton causes a defect where one cation is doubly protonated and another deprotonated. Figure 8c illustrates a hydrogen-bonded chain where the proton is placed approximately in the middle of two molecular species.

**Figure 9:**
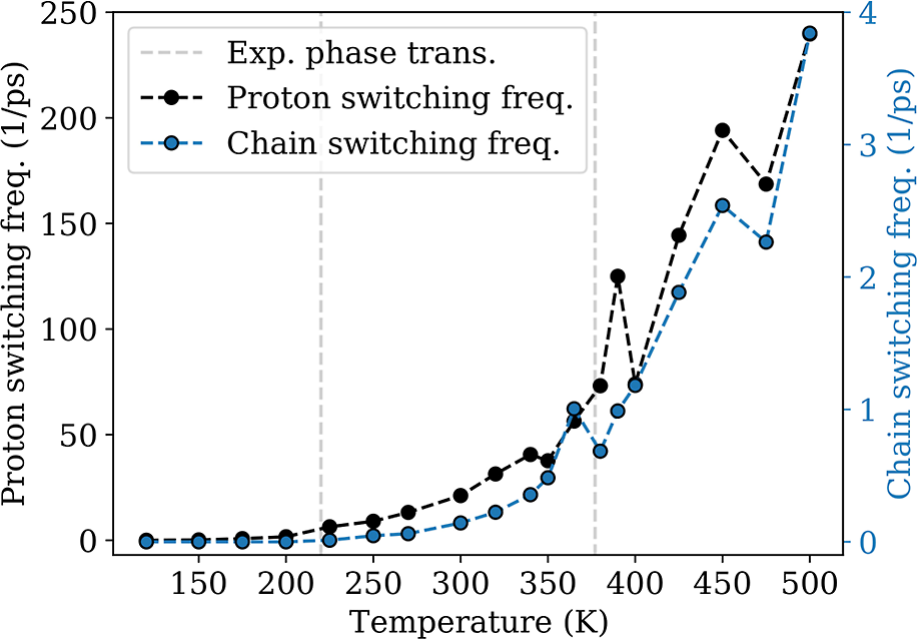
Frequency of proton transfer in HdabcoClO_4_. Protons are transferred at all temperatures, and the switching frequency of the orientation of hydrogen-bonded chains of Hdabco^+^-cations.

**Figure 10:**
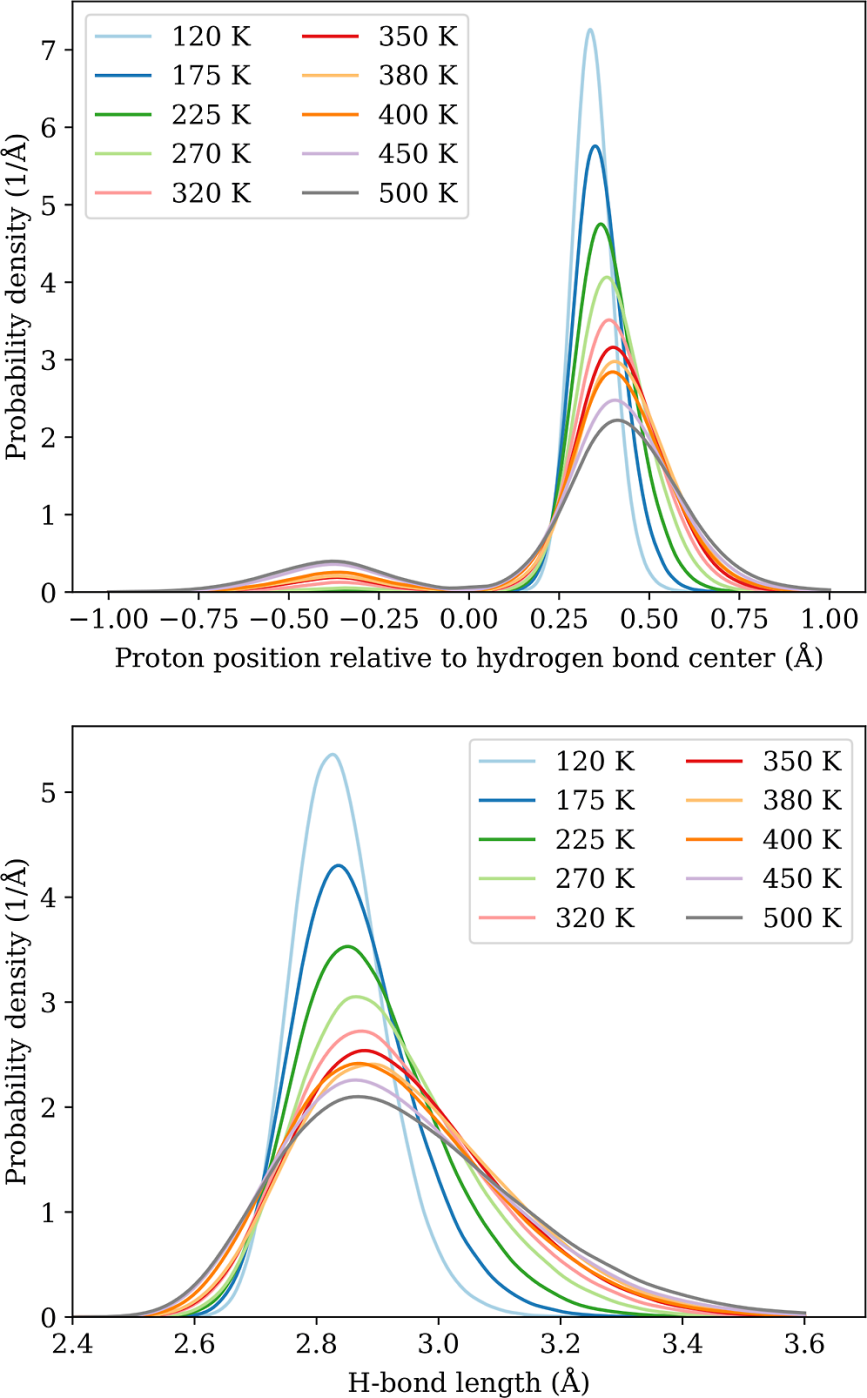
Probability density of the proton positions relative to the center of their hydrogen bond (top), and the corresponding hydrogen bond lengths between Hdabco^+^-cations (bottom). Positive proton position values indicate that the proton is oriented in the same direction as the hydrogen-bonded chain of Hdabco^+^-cations, while negative indicate the opposite.

Such defects are observable already at temperatures of 150 K and above. In Figure 9, the black curve shows the frequencies of protons switching between two neighbor Hdabco^+^-cations. The plot shows two changes in slope, one at 225 and one around 365 K. The blue curve shows the switching frequency of the orientation of the hydrogen-bonded chains. The trend is similar to the proton transfer frequencies, but the switching frequency of a whole chain is 2 orders of magnitude lower. This shows that most proton transfer events create short-lived local defects. Figure 10 (top panel) plots the proton position relative to the middle of its hydrogen bond. Here, positive values reflect protons in hydrogen bonds that are oriented in the same direction as the overall orientation of its hydrogen-bonded chain. Negative values indicate that the proton is in a hydrogen bond with an opposite orientation relative to the chain. The plot shows a higher probability of finding protons aligned with the chain than ones that do not align with the chain directions at all temperatures This preference shows that there is still a directionality of the hydrogen-bonded chains at elevated temperatures and not a full disorder of protons. Similar, in the bottom panel of Figure 10 shows the distribution of the lengths of hydrogen bonds between Hdabco^+^ cations, showing a slight increase in most typical bond lengths, but also larger fluctuations in the bond lengths as temperature increases.

While this study provides qualitative insight into proton transfer in organic systems that may carry over to other organic and hybrid crystals, DFT computations of proton transfer barriers are very sensitive to the exchange–correlation functional employed. Seyedraoufi and Berland^77^ recently found for a set of molecular dimers that while vdW-DF correlation can significantly improve proton transfer barriers compared to using correlation at the generalized gradient approximation (GGA), which severely underestimated barrier heights, the vdW-DF-cx variant also underestimates such barriers, due to its “soft” exchange form,^56,78^ Using vdW-DF2,^79^ which predicted more accurate barriers could thus have improved the accuracy for this system, and so would adopting a hybrid functional, such as the vdW-DF-cx0-20 functional.^80,81^ Such a functional would have likely significantly delayed the onset of proton transfer.

### Summary of Phase Transitions Parameters

A summary of computed phase transition characteristics of HdabcoClO_4_ are listed in Table 4, alongside the experimental phase transition temperatures.^44^ The computational results are consistent with experimental measurements overall. In the transition from III to II, neither theory nor experiment found any marked changes in volume; although in the computations, we found changes in the lattice constants as seen in Figure 2. The reduction in displacement of Hdabco^+^-cations relative to ClO_4_^–^-anions at 200 K, as shown in Figure 3 is also indicative of a phase transition between these two ferroelectric phases, and so is the onset of orientation disorder between 200 and 225 K, as evidenced by Figures 4 and 5. Moreover, the MD data in Figures 4 and 6 is in line with the increased thermal vibration reported for this phase.^18^ The clear slope change in line with proton disorder in phase II at 200 K, is also in line with a phase transition, but experimentally proton disorder has only been observed in phase III.^18^

**Table 4:** Experimental Phase Transition Temperatures^44^ and an Overview of the Onset of Disorder and symmetry Changes in the MLFF Simulations of HdabcoClO_4_

	III to II (K)	II to I (K)
experimental phase trans.	220	377
volume expansion		380
ferro. displacement	200	380–450
ClO_4_^–^ disorder	225	380
Hdabco^+^ disorder		380
H-bond chain switching	225	365

For the phase transition between II and I, similar features to those in the experiment are found, albeit spread at different temperatures. The autocorrelation of ClO_4_^–^ (Figure 5) indicates the onset of essentially free rotation of this species at 380 K, in line with experiment. Moreover, at this temperature, we also find the onset of molecular rotations of Hdabco^+^. The change in sublattice displacement (Figure 3) is less clear, showing increasing deviations from temperatures of 380 K and above, while reaching values close to zero first at 450 K. This apparent disparity with experiments may also hint at nanoscale domain formation, i.e., using much larger supercells and longer time runs might average out to provide a cubic unit cell in line with experiment structure characterization.

## Conclusion and Outlook

An MLFF was trained for HdabcoClO_4_ using the neural network NeuralIL, with an active learning procedure to diversify the training set. Our study highlights how MLFF-based MD can be used to gain fundamental insight into the dynamical properties of plastic ionic crystals, with overall encouraging agreement between computed and measured phase transition properties. By using a fully ab initio approach that requires no knowledge of predefined bonding properties, our study highlights how MLFF can be used both for computational design and analysis of the emerging class of dynamical materials such as plastic ionic crystals, in particular in combination with advanced structural characterization methods. However, the disparities between theory and experiment also highlight the need for systematic benchmarking of both MLFF approaches and DFT exchange–correlation functionals for out-of-equilibrium geometries for systems exhibiting complex noncovalent bonding, such as plastic ionic crystals.

## Data Availability

All training data can be accessed through the Nomad database with DOI: 10.17172/NOMAD/2024.09.19-1.

## References

[ref1] Pringle J. M. Recent progress in the development and use of organic ionic plastic crystal electrolytes. Phys. Chem. Chem. Phys. 2013, 15 (5), 1339–1351. 10.1039/C2CP43267F. 23184152

[ref2] Armel V.; et al. Organic ionic plastic crystal electrolytes; a new class of electrolyte for high efficiency solid state dye-sensitized solar cells. Energy Environ. Sci. 2011, 4 (6), 2234–2239. 10.1039/c1ee01062j.

[ref3] Zhu H.; et al. Organic Ionic Plastic Crystals as Solid-State Electrolytes. Trends Chem. 2019, 1 (1), 126–140. 10.1016/j.trechm.2019.01.002.

[ref4] Salgado-Beceiro J.; Bermúdez-García J. M.; Stern-Taulats E.; García-Ben J.; Castro-García S.; Sánchez-Andújar M.; Moya X.; Señarís-Rodríguez M. A. Hybrid ionic plastic crystals in the race for enhanced low-pressure barocaloric materials. ChemRxiv 2021, 10.26434/chemrxiv-2021-c4hx5.

[ref5] Harada J. Plastic/ferroelectric molecular crystals: Ferroelectric performance in bulk polycrystalline forms. APL Mater. 2021, 9 (2), 020901 10.1063/5.0039066.

[ref6] Das S.; Mondal A.; Reddy C. M. Harnessing molecular rotations in plastic crystals: a holistic view for crystal engineering of adaptive soft materials. Chem. Soc. Rev. 2020, 49 (24), 8878–8896. 10.1039/D0CS00475H. 33185234

[ref7] Shi P.-P.; Tang Y. Y.; Li P. F.; Liao W. Q.; Wang Z. X.; Ye Q.; Xiong R. G. Symmetry breaking in molecular ferroelectrics. Chem. Soc. Rev. 2016, 45, 3811–3827. 10.1039/c5cs00308c. 27051889

[ref8] Pan Q.; Xiong Y. A.; Sha T. T.; You Y. M. Recent progress in the piezoelectricity of molecular ferroelectrics. Mater. Chem. Front. 2021, 5, 44–59. 10.1039/d0qm00288g.

[ref9] Walker J.; et al. Electromechanical properties of uniaxial polar ionic plastic crystal [(C _2_ H _5_) _4_ N][FeBrCl _3_ ]. J. Phys. Energy 2024, 6 (2), 025026 10.1088/2515-7655/ad405c.

[ref10] Walker J.; Scherrer S.; Løndal N. S.; Grande T.; Einarsrud M. A. Electric field dependent polarization switching of tetramethylammonium bromotrichloroferrate(III) ferroelectric plastic crystals. Appl. Phys. Lett. 2020, 116, 242902 10.1063/5.0004387.

[ref11] Jin L.; et al. An organic ionic plastic crystal electrolyte for rate capability and stability of ambient temperature lithium batteries. Energy Environ. Sci. 2014, 7 (10), 3352–3361. 10.1039/C4EE01085J.

[ref12] Owczarek M.; Hujsak K. A.; Ferris D. P.; Prokofjevs A.; Majerz I.; Szklarz P.; Zhang H.; Sarjeant A. A.; Stern C. L.; Jakubas R.; et al. Flexible ferroelectric organic crystals. Nat. Commun. 2016, 7 (1), 13108 10.1038/ncomms13108. 27734829 PMC5065626

[ref13] Deng S.; et al. A novel ferroelectric based on quinuclidine derivatives. Chin. Chem. Lett. 2020, 31 (6), 1686–1689. 10.1016/j.cclet.2019.11.011.

[ref14] Lan X.; et al. Cation and Anion Transfer in Quinuclidinium Hexafluorophosphate Plastic Crystal: Role of Constituent Ions and the Crystalline Structure. J. Phys. Chem. C 2021, 125 (38), 21169–21178. 10.1021/acs.jpcc.1c05891.

[ref15] Harada J.; Shimojo T.; Oyamaguchi H.; Hasegawa H.; Takahashi Y.; Satomi K.; Suzuki Y.; Kawamata J.; Inabe T. Directionally tunable and mechanically deformable ferroelectric crystals from rotating polar globular ionic molecules. Nat. Chem. 2016, 8, 946–952. 10.1038/nchem.2567. 27657871

[ref16] Timmermans J. Plastic crystals: A historical review. J. Phys. Chem. Solids 1961, 18 (1), 1–8. 10.1016/0022-3697(61)90076-2.

[ref17] Mondal A.; et al. Metal-like Ductility in Organic Plastic Crystals: Role of Molecular Shape and Dihydrogen Bonding Interactions in Aminoboranes. Angew. Chem., Int. Ed. Engl. 2020, 59 (27), 10971–10980. 10.1002/anie.202001060. 32087039

[ref18] Olejniczak A.; Szafrański M.; Katrusiak A. Pressure–Temperature Phase Diagrams and Transition Mechanisms of Hybrid Organic–Inorganic NH-N Bonded Ferroelectrics. Cryst. Growth Des. 2018, 18 (11), 6488–6496. 10.1021/acs.cgd.8b00581.

[ref19] Yoneya M.; Harada J. Molecular Dynamics Simulation Study of the Plastic/Ferroelectric Crystal Quinuclidinium Perrhenate. J. Phys. Chem. C 2020, 124 (3), 2171–2177. 10.1021/acs.jpcc.9b09559.

[ref20] Li B.; Kawakita Y.; Ohira-Kawamura S.; Sugahara T.; Wang H.; Wang J.; Chen Y.; Kawaguchi S. I.; Kawaguchi S.; Ohara K.; et al. Colossal barocaloric effects in plastic crystals. Nature 2019, 567, 506–510. 10.1038/s41586-019-1042-5. 30918372

[ref21] Liu Y.; Zhou H.; Xu Z.; Liu D.; Li J.; Hu F.; Ma T. Giant barocaloric effect in neopentylglycol-graphene nanosheets composites with large thermal conductivity. Mater. Res. Lett. 2022, 10, 675–681. 10.1080/21663831.2022.2086442.

[ref22] Aznar A.; et al. Reversible and irreversible colossal barocaloric effects in plastic crystals. J. Mater. Chem. A 2020, 8 (2), 639–647. 10.1039/C9TA10947A.

[ref23] Li F. B.; Li M.; Xu X.; Yang Z. C.; Xu H.; Jia C. K.; Li K.; He J.; Li B.; Wang H. Understanding colossal barocaloric effects in plastic crystals. Nat. Commun. 2020, 11 (1), 4190 10.1038/s41467-020-18043-1. 32826887 PMC7442785

[ref24] Lloveras P.; Aznar A.; Barrio M.; Negrier P.; Popescu C.; Planes A.; Mañosa L.; Stern-Taulats E.; Avramenko A.; Mathur N. D.; et al. Colossal barocaloric effects near room temperature in plastic crystals of neopentylglycol. Nat. Commun. 2019, 10 (1), 1803 10.1038/s41467-019-09730-9. 31000715 PMC6472423

[ref25] Tilley R. J. D. Insulating Solids. In: Understanding solids the science of materials, 2nd ed.; Wiley, 2013; pp 327–354

[ref26] Harada J.; et al. Ferroelectricity and Piezoelectricity in Free-Standing Polycrystalline Films of Plastic Crystals. J. Am. Chem. Soc. 2018, 140 (1), 346–354. 10.1021/jacs.7b10539. 29224333

[ref27] Harada J.; et al. “Plastic/Ferroelectric Crystals with Easily Switchable Polarization: Low-Voltage Operation, Unprecedentedly High Pyroelectric Performance, and Large Piezoelectric Effect in Polycrystalline Forms”. J. Am. Chem. Soc 2019, 141 (23), 9349–9357. 10.1021/jacs.9b03369. 31184147

[ref28] González-Izquierdo P.; et al. ((R)-(−)-3-Hydroxyquinuclidium)[FeCl4]; a plastic hybrid compound with chirality, ferroelectricity and long range magnetic ordering. J. Mater. Chem. C 2021, 9 (13), 4453–4465. 10.1039/D0TC05800A.

[ref29] Seyedraoufi S.; et al. Database mining and first-principles assessment of organic proton-transfer ferroelectrics. Phys. Rev. Mater. 2024, 8 (5), 054413 10.1103/PhysRevMaterials.8.054413.

[ref30] Dypvik Sødahl E.; Seyedraoufi S.; Görbitz C. H.; Berland K. Ferroelectric Crystals of Globular Molecules: Cambridge Structural Database Mining and Computational Assessment. Cryst. Growth Des. 2023, 23, 8607–8619. 10.1021/acs.cgd.3c00713.

[ref31] González M. Force fields and molecular dynamics simulations. Collection SFN 2011, 169–200. 10.1051/SFN/201112009.

[ref32] Unke O. T.; Chmiela S.; Sauceda H. E.; Gastegger M.; Poltavsky I.; Schütt K. T.; Tkatchenko A.; Müller K. R. Machine Learning Force Fields. Chem. Rev. 2021, 121, 10142–10186. 10.1021/acs.chemrev.0c01111. 33705118 PMC8391964

[ref33] Car R. Introduction to Density-Functional Theory and ab-Initio Molecular Dynamics. Quant. Struct.-Act. Relat. 2002, 21 (2), 97–104. 10.1002/1521-3838(200207)21:2<97::AID-QSAR97>3.0.CO;2-6.

[ref34] Wieser S.; Zojer E. Machine learned force-fields for an Ab-initio quality description of metal-organic frameworks. npj Comput. Mater. 2024, 10 (1), 18 10.1038/s41524-024-01205-w.

[ref35] Friederich P.; et al. Machine-learned potentials for next-generation matter simulations. Nat. Mater. 2021, 20 (6), 750–761. 10.1038/s41563-020-0777-6. 34045696

[ref36] Carrete J.; Montes-Campos H.; Wanzenböck R.; Heid E.; Madsen G. K. H. Deep ensembles vs committees for uncertainty estimation in neural-network force fields: Comparison and application to active learning. J. Chem. Phys. 2023, 158, 204801 10.1063/5.0146905. 37212411

[ref37] Wu S.; et al. Applications and Advances in Machine Learning Force Fields. J. Chem. Inf. Model. 2023, 63 (22), 6972–6985. 10.1021/acs.jcim.3c00889. 37751546

[ref38] Chmiela S.; Tkatchenko A.; Sauceda H. E.; Poltavsky I.; Schütt K. T.; Müller K. R. Machine learning of accurate energy-conserving molecular force fields. Sci. Adv. 2017, 3 (5), e1603015 10.1126/sciadv.1603015. 28508076 PMC5419702

[ref39] Schleder G. R.; et al. From DFT to machine learning: recent approaches to materials science–a review. J. Phys. Mater. 2019, 2 (3), 032001 10.1088/2515-7639/ab084b.

[ref40] Fiedler L.; et al. Deep dive into machine learning density functional theory for materials science and chemistry. Phys. Rev. Mater. 2022, 6 (4), 040301 10.1103/PhysRevMaterials.6.040301.

[ref41] Tang Y.-Y.; Li P. F.; Shi P. P.; Zhang W. Y.; Wang Z. X.; You Y. M.; Ye H. Y.; Nakamura T.; Xiong R. G. Visualization of Room-Temperature Ferroelectricity and Polarization Rotation in the Thin Film of Quinuclidinium Perrhenate. Phys. Rev. Lett. 2017, 119, 207602 10.1103/physrevlett.119.207602. 29219370

[ref42] Tang Y.-Y.; Xie Y.; Ai Y.; Liao W. Q.; Li P. F.; Nakamura T.; Xiong R. G. Organic Ferroelectric Vortex–Antivortex Domain Structure. J. Am. Chem. Soc. 2020, 142, 21932–21937. 10.1021/jacs.0c11416. 33326208

[ref43] Tang Y.-Y.; et al. Ultrafast Polarization Switching in a Biaxial Molecular Ferroelectric Thin Film: [Hdabco]ClO _4_. J. Am. Chem. Soc. 2016, 138 (48), 15784–15789. 10.1021/jacs.6b10595. 27934003

[ref44] Olejniczak A.; Anioła M.; Szafrański M.; Budzianowski A.; Katrusiak A. New Polar Phases of 1,4-Diazabicyclo[2.2.2]octane Perchlorate, An NH ^+^ ··· N Hydrogen-Bonded Ferroelectric. Cryst. Growth Des. 2013, 13, 2872–2879. 10.1021/cg400276c.

[ref45] Li W.; Tang G.; Zhang G.; Jafri H. M.; Zhou J.; Liu D.; Liu Y.; Wang J.; Jin K.; Hu Y.; et al. Improper molecular ferroelectrics with simultaneous ultrahigh pyroelectricity and figures of merit. Sci. Adv. 2021, 7 (5), eabe3068 10.1126/sciadv.abe3068. 33514555 PMC7846162

[ref46] Katrusiak A.; Szafrański M. Ferroelectricity in NH-N Hydrogen Bonded Crystals. Phys. Rev. Lett. 1999, 82, 576–579. 10.1103/PhysRevLett.82.576.

[ref47] Katrusiak A. Proton dynamics in NH–N hydrogen bond in the paraelectric structure of 1,4-diazabicyclo[2.2.2]octane perchlorate. J. Mol. Struct. 2000, 552 (1), 159–164. 10.1016/S0022-2860(00)00475-0.

[ref48] Blöchl P. E. Projector augmented-wave method. Phys. Rev. B 1994, 50.24, 17953–17979. 10.1103/physrevb.50.17953. 9976227

[ref49] Kresse G.; Joubert D. From ultrasoft pseudopotentials to the projector augmented-wave method. Phys. Rev. B 1999, 59 (3), 1758–1775. 10.1103/PhysRevB.59.1758.

[ref50] Kresse G.; Hafner J. Ab initio molecular-dynamics simulation of the liquid-metal–amorphous-semiconductor transition in germanium. Phys. Rev. B 1994, 49, 14251–14269. 10.1103/physrevb.49.14251. 10010505

[ref51] Kresse G.; Hafner J. Ab initio molecular dynamics for liquid metals. Phys. Rev. B 1993, 47 (1), 558–561. 10.1103/PhysRevB.47.558. 10004490

[ref52] Kresse G.; Furthmüller J. Efficiency of ab-initio total energy calculations for metals and semiconductors using a plane-wave basis set. Comput. Mater. Sci. 1996, 6 (1), 15–50. 10.1016/0927-0256(96)00008-0.

[ref53] Kresse G.; Furthmüller J. Efficient iterative schemes for ab initio total-energy calculations using a plane-wave basis set. Phys. Rev. B 1996, 54 (16), 11169–11186. 10.1103/PhysRevB.54.11169. 9984901

[ref54] Berland K.; et al. Van der Waals forces in density functional theory: a review of the vdW-DF method. Rep. Prog. Phys. 2015, 78 (6), 066501 10.1088/0034-4885/78/6/066501. 25978530

[ref55] Berland K.; Hyldgaard P. Exchange functional that tests the robustness of the plasmon description of the van der Waals density functional. Phys. Rev. B 2014, 89 (3), 035412 10.1103/PhysRevB.89.035412.

[ref56] Berland K.; Arter C. A.; Cooper V. R.; Lee K.; Lundqvist B. I.; Schröder E.; Thonhauser T.; Hyldgaard P. van der Waals density functionals built upon the electron-gas tradition: Facing the challenge of competing interactions. J. Chem. Phys. 2014, 140 (18), 18A539 10.1063/1.4871731. 24832347

[ref57] Tran F.; et al. Nonlocal van der Waals functionals for solids: Choosing an appropriate one. Phys. Rev. Mater. 2019, 3 (6), 063602 10.1103/PhysRevMaterials.3.063602.

[ref58] Chakraborty D.; Berland K.; Thonhauser T. Next-Generation Nonlocal van der Waals Density Functional. J. Chem. Theory Comput. 2020, 16 (9), 5893–5911. 10.1021/acs.jctc.0c00471. 32786912

[ref59] Sødahl E. D.; Walker J.; Berland K. Piezoelectric Response of Plastic Ionic Molecular Crystals: Role of Molecular Rotation. Cryst. Growth Des. 2023, 23 (2), 729–740. 10.1021/acs.cgd.2c00854.

[ref60] Nosé S. A unified formulation of the constant temperature molecular dynamics methods. J. Chem. Phys. 1984, 81 (1), 511–519. 10.1063/1.447334.

[ref61] Hoover W. G. Canonical dynamics: Equilibrium phase-space distributions. Phys. Rev. A 1985, 31 (3), 1695–1697. 10.1103/PhysRevA.31.1695. 9895674

[ref62] He K. “Deep Residual Learning for Image Recognition”. 2016 IEEE Conference on Computer Vision and Pattern Recognition (CVPR); IEEE, 2016; pp 770–778.

[ref63] Bradbury J.; JAX: composable transformations of Python+NumPy programs, Version 0.3.13, 2018.http://github.com/google/jax.

[ref64] Heek J.; Flax: A neural network library and ecosystem for JAX, Version 0.9.0, 2024. http://github.com/google/flax.

[ref65] Montes-Campos H.; et al. A Differentiable Neural-Network Force Field for Ionic Liquids. J. Chem. Inf. Model. 2022, 62 (1), 88–101. 10.1021/acs.jcim.1c01380. 34941253 PMC8757435

[ref66] Metz L.; et al. VeLO: Training Versatile Learned Optimizers by Scaling Up. arXiv 2022, arXiv:2211.09760 10.48550/arXiv.2211.09760.

[ref67] Schoenholz S. S.; Cubuk E. D. “JAX, M.D. A framework for differentiable physics*”. J. Stat. Mech.:Theory Exp. 2021, 2021 (12), 124016 10.1088/1742-5468/ac3ae9.

[ref68] Martyna G. J.; Klein M. L.; Tuckerman M. Nosé–Hoover chains: The canonical ensemble via continuous dynamics. J. Chem. Phys. 1992, 97 (4), 2635–2643. 10.1063/1.463940.

[ref69] Martyna G. J.; Tobias D. J.; Klein M. L. Constant pressure molecular dynamics algorithms. J. Chem. Phys. 1994, 101 (5), 4177–4189. 10.1063/1.467468.

[ref70] Yu T.-Q.; et al. Measure-preserving integrators for molecular dynamics in the isothermal–isobaric ensemble derived from the Liouville operator. Chem. Phys. 2010, 370 (1), 294–305. 10.1016/j.chemphys.2010.02.014.

[ref71] Bichelmaier S. Ab-initio modelling of material properties using elements of artificial intelligence, 2023. [Dissertation, Technische Universitat Wien]. reposiTUm. PhD dissertation

[ref72] Berland K.; Chakraborty D.; Thonhauser T. van der Waals density functional with corrected *C*_6_ coefficients. Phys. Rev. B 2019, 99 (19), 195418 10.1103/PhysRevB.99.195418.

[ref73] Morana M.; et al. Cubic or Not Cubic? Combined Experimental and Computational Investigation of the Short-Range Order of Tin Halide Perovskites. J. Phys. Chem. Lett. 2023, 14 (8), 2178–2186. 10.1021/acs.jpclett.3c00105. 36808992 PMC9986956

[ref74] Krbal M.; et al. Local atomic order of crystalline Ge_8_Sb_2_Te_11_ across the ferroelectric to paraelectric transition: The role of vacancies and static disorder. Phys. Rev. B 2011, 84 (10), 104106 10.1103/PhysRevB.84.104106.

[ref75] Reuveni G.; et al. Static and Dynamic Disorder in Formamidinium Lead Bromide Single Crystals. J. Phys. Chem. Lett. 2023, 14 (5), 1288–1293. 10.1021/acs.jpclett.2c03337. 36722023 PMC9923750

[ref76] Adebahr J.; et al. Structure and dynamics of the plastic crystal tetramethylammonium dicyanamide—a molecular dynamics study. Solid State Ionics 2006, 177, 2845–2850. 10.1016/j.ssi.2006.07.061.

[ref77] Seyedraoufi S.; Berland K. Improved proton-transfer barriers with van der Waals density functionals: Role of repulsive non-local correlation. J. Chem. Phys. 2022, 156 (24), 244106 10.1063/5.0095128. 35778093

[ref78] Jenkins T.; Berland K.; Thonhauser T. Reduced-gradient analysis of van der Waals complexes. Electronic Structure 2021, 3 (3), 034009 10.1088/2516-1075/ac25d7.

[ref79] Lee K.; et al. Higher-accuracy van der Waals density functional. Phys. Rev. B 2010, 82 (8), 081101 10.1103/PhysRevB.82.081101.

[ref80] Berland K.; Jiao Y.; Lee J. H.; Rangel T.; Neaton J. B.; Hyldgaard P. Assessment of two hybrid van der Waals density functionals for covalent and non-covalent binding of molecules. J. Chem. Phys. 2017, 146, 23 10.1063/1.4986522. 28641426

[ref81] Jiao Y.; Schröder E.; Hyldgaard P. Extent of Fock-exchange mixing for a hybrid van der Waals density functional?. J. Chem. Phys. 2018, 148 (19), 194115 10.1063/1.5012870. 30307250

